# Production of trehalose with trehalose synthase expressed and displayed on the surface of *Bacillus subtilis* spores

**DOI:** 10.1186/s12934-019-1152-7

**Published:** 2019-06-03

**Authors:** Hongling Liu, Shaojie Yang, Xihui Wang, Tengfei Wang

**Affiliations:** 1 State Key Laboratory of Biobased Material and Green Papermaking (LBMP), Qilu University of Technology (Shandong Academy of Sciences), No. 3501, University Road, Changqing District, Jinan, 250353 Shandong People’s Republic of China; 2 Key Laboratory of Shandong Microbial Engineering, College of Bioengineering, Qilu University of Technology (Shandong Academy of Sciences), Jinan, 250353 Shandong People’s Republic of China; 3 Key Laboratory of Industrial Fermentation Microbiology, Tianjin University of Science & Technology, Ministry of Education, Tianjin, 300457 People’s Republic of China; 4 Tianjin Key Lab of Industrial Microbiology, Tianjin University of Science and Technology, Tianjin, 300457 People’s Republic of China

**Keywords:** Spore surface display, Trehalose synthase, Gene knockout, *Bacillus subtilis* WB800n

## Abstract

**Background:**

*Bacillus subtilis* spores have been commonly used for the surface display of various food-related or human antigens or enzymes. For successful display, the target protein needs to be fused with an anchor protein. The preferred anchored proteins are the outer-coat proteins of spores; outer-coat proteins G (CotG) and C (CotC) are commonly used. In this study, mutant trehalose synthase (V407M/K490L/R680E TreS) was displayed on the surface of *B. subtilis* WB800n spores using CotG and CotC individually or in combination as an anchoring protein.

**Results:**

Western blotting, immunofluorescence, dot blot, and enzymatic-activity assays detected TreS on the spore surface. The TreS activity with CotC and CotG together as the anchor protein was greater than the sum of the enzymatic activities with CotC or CotG alone. The TreS displayed on the spore surface with CotC and CotG together as the anchoring protein showed elevated and stable specific activity. To ensure spore stability and prevent spore germination in the trehalose preparation system, two germination-specific lytic genes, *sleB* and *cwlJ*, were deleted from the *B. subtilis* WB800n genome. It was demonstrated that this deletion did not affect the growth and spore formation of *B. subtilis* WB800n but strongly inhibited germination of the spores during transformation. The conversion rate of trehalose from 300 g/L maltose by *B. subtilis* strain WB800n(*ΔsleB*, *ΔcwlJ*)/*cotC*-*treS*–*cotG*-*treS* was 74.1% at 12 h (350 U/[g maltose]), and its enzymatic activity was largely retained, with a conversion rate of 73% after four cycles.

**Conclusions:**

The spore surface display system based on food-grade *B. subtilis* with CotC and CotG as a combined carrier appears to be a powerful technology for TreS expression, which may be used for the biotransformation of d-maltose into d-trehalose.

**Electronic supplementary material:**

The online version of this article (10.1186/s12934-019-1152-7) contains supplementary material, which is available to authorized users.

## Background

Trehalose is a stable and useful nonreducing disaccharide composed of two glucose molecules linked by an α,α-1,1-glycosidic bond. Trehalose is widespread among yeasts (and other fungi), bacteria, invertebrates, plants, and insects [1]. It can serve as a carbon source reserve and a component of the cell wall in some organisms [2–4] and as a protectant, sweetener, stabilizing agent, and moisture retention agent in the production of foodstuffs, pharmaceuticals, cosmetics, and agricultural products [1, 5–10].

Trehalose synthase (TreS) catalyzes the reversible interconversion of maltose and trehalose [11]. TreS is mainly expressed by bacteria. The TreS pathway is attractive for the production of trehalose owing to the low cost of the substrate, simplicity of the reaction, and a high conversion yield [1, 11]. The *treS* genes of different bacteria have been cloned. As early as 1995, TreS was discovered in (and purified from) *Pimelobacter* sp. R48 [11]. Shortly afterwards, the *treS* gene from *Pimelobacter* sp. R48 and thermophilic aquatic bacteria was obtained by screening of a genomic DNA library with a radioprobe [12]. Subsequently, *treS* from *Mycobacterium tuberculosis* was cloned and heterologously expressed in *Escherichia coli*, and the optimum reaction conditions were studied [13]. Additionally, the *treS* gene from *Pseudomonas stutzeri* CJ38 was obtained via screening of its genomic library using malPQ-deficient strains and the MacConkey medium; this experiment showed that *treS* from *P. stutzeri* CJ38 does not generate glucose byproducts during its reaction and is suitable for industrial production of trehalose [14]. Meanwhile, *treS* from *Picrophilus torridus*, a dry-thermophilic acidophilic bacterium, is resistant to acidity and high temperatures and still has high activity at pH 5.0 and 60 °C [15]. In addition, *treS* has been found in *Arthrobacter aurescens* [16], *Enterobacter hormaechei* [17], *Meiothermus ruber* [18], and *Pseudomonas putida* [19], and these genes can be stably overexpressed in *E. coli*. To recover TreS from *E. coli*, it is necessary to disrupt the bacterial cells, and this procedure increases production costs. Furthermore, an *E. coli* endotoxin released during the cell lysis may contaminate the finished product.

*Bacillus subtilis* is a spore-forming bacterium. A technology that can decorate the surface of the spores with various molecules was proposed in 2001. In this method, heterologous proteins are immobilized on the exterior of spores [20, 21]. The spore surface display system has since become an attractive option for various purposes. To construct this system, outer-coat proteins of spores are preferred. Outer-coat proteins including CotB [20, 22, 23], CotG [24–26], and CotC [27, 28] have been successfully used as anchoring motifs to display various heterologous proteins on the surface of *B. subtilis* spores. Enzymes anchored on the spore surface are particularly suitable for repeated-batch fermentation because they facilitate biomass recovery for the use in subsequent batches. Biocatalysts in the form of whole cells have advantages over purified enzymes in many industrial bioconversion processes. Intracellular enzymes extracted from cells can be used in solution for only one batch process if they are not immobilized. This shortcoming can be eliminated by enzyme immobilization; however, such preparations are frequently not sufficiently stable, and their productivity is usually unsatisfactory for industrial purposes. These disadvantages can be overcome by surface anchoring of the desired proteins on spores.

Spore germination is defined as the event leading to a loss of spore-specific properties that requires degradation of the thick cortical peptidoglycan by germination-specific cortex-lytic enzymes [29]. In *B. subtilis*, two key cortex-lytic enzymes, namely CwlJ and SleB, have been uncovered [30]. CwlJ is located at the cortex–coat junction, whereas SleB is present in both outer and inner spore membranes and is more resistant to heat and humidity than CwlJ [30]. Cortex hydrolysis leads to complete rehydration of the spore core. It has been reported that the germinating spores without CwlJ show delays in the light density loss and cortical peptidoglycan release, whereas the absence of SleB also delays the release of cortical debris [31]. A double mutant lacking both SleB and CwlJ is blocked during cortical hydrolysis and progresses through outgrowth to produce colonies at a frequency 1000-fold lower than that of wild-type strains [31].

In the present study, for the first time, a mutant TreS (V407M/K490L/R680E) was successfully displayed on the surface of *B. subtilis* spores using proteins CotC and CotG either alone or together as an anchoring protein. The system was successfully applied to the biotransformation of d-maltose into d-trehalose.

## Results

### Construction of recombinant plasmids and bacterial strains

CotC and CotG, which are morphogenetic proteins located on the spore crust, were utilized individually or in combination as the anchoring protein to enable the display of the mutant TreS (V407M/K490L/R680E) on the surface of *B. subtilis* WB800n spores. After we obtained recombinant plasmids pDG1730-cotC-treS, pDG1730-cotG-treS, and pDG1730-cotC-treS–cotG-treS, restriction digestion and sequencing were performed. The sizes of the obtained DNA fragments matched the expected lengths of cotC-treS and cotG-treS. The linearized pDG1730-cotC-treS, pDG1730-cotG-treS, and pDG1730-cotC-treS–cotG-treS plasmids were electroporated into competent *B. subtilis* WB800n cells, and individual clones resulting from each transformation were examined by PCR.

**Fig. 1:**
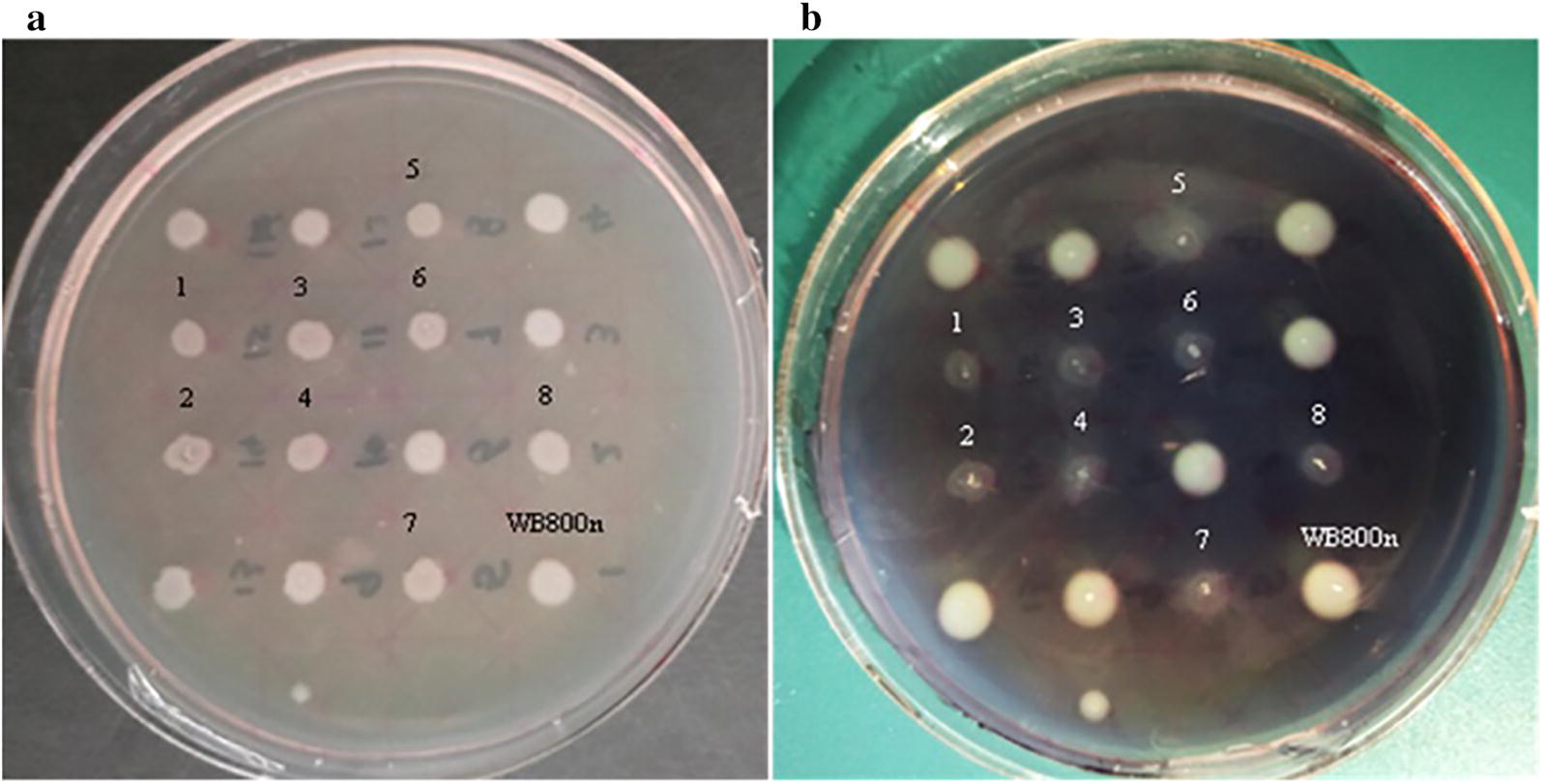
Validation of recombinant *B. subtilis* WB800n using starch agar. 1 and 2: *B. subtilis* WB800n/*cotC*-*treS*; 3 and 4: *B. subtilis* WB800n/*cotG*-*treS*; 5–8: *B. subtilis* WB800n/*cotC*-*treS*–*cotG*-*treS*. **a** Before iodine addition, **b** after iodine addition

To ensure that TreS is localized to the surface of *B. subtilis* WB800n spores, recombinant versions of the strain carrying gene *cotC*-*treS*, *cotG*-*treS*, or *cotC*-*treS*–*cotG*-*treS* were generated. In these recombinants, the *cotC*-*treS*, *cotG*-*treS*, or *cotC*-*treS*–*cotG*-*treS* fusion gene was introduced at the *amyE* (the α-amylase [Amy]-encoding gene) locus of the *B. subtilis* WB800n chromosome by double cross-over recombination. Because introduction of the gene at the *amyE* locus was expected to yield an amylase-negative phenotype on Luria–Bertani (LB) plates containing 1% starch, integration-positive clones with one of the fusion genes integrated at the *amyE* locus of the *B. subtilis* WB800n chromosome were identified by appearance of a blue color after staining with iodine. The control, in which there was no integration into the *B. subtilis* WB800n chromosome, did not develop the blue color because the expression of *amyE* resulted in hydrolysis of the starch on the plate. After the *B. subtilis* WB800n and recombinant bacteria were grown on LB plates containing 1% starch for 12 h at 37 °C (Fig. 1a), iodine was added to detect the amylase activity, which manifested itself via the emergence of transparent plaques. The perimeter of *B. subtilis* WB800n colonies contained an obvious area of starch hydrolysis, and such a transparent area was absent around colonies of the recombinant bacteria, indicating the successful integration of the target gene at the chromosomal *amyE* locus (Fig. 1b).

### Identification of Cot-treS displayed on the spore surface of recombinant *B. subtilis* WB800n

**Fig. 2:**
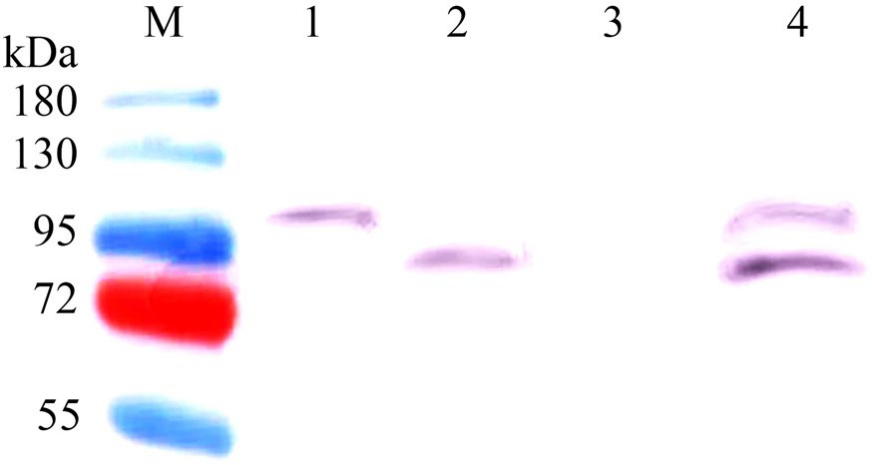
Western blot analysis of TreS displayed on the spore surface. Spores were incubated with the mouse anti-His tag antibody (dilution 1:3000), followed by probing with a horseradish peroxidase-conjugated goat anti-mouse IgG antibody (1:2000). Lane M: markers; lane 1: CotG-TreS; lane 2: CotC-TreS; lane 3: *B. subtilis* WB800n control; lane 4: CotC-TreS–CotG-TreS

To confirm the expression of the CotC-TreS and CotG-treS fusion proteins on the spore surface, an anti-His-tag antibody was employed for western blot analysis. The theoretical weight of the CotC-TreS and CotG-TreS fusion proteins including the His-tag was 99.5 and 84.4 kDa, respectively, suggesting that the *cotC*-*treS* and *cotG*-*treS* fusion genes were successfully expressed heterologously. The apparent size of the fusion proteins detected in the extracts of the recombinant spores was in good agreement with the theoretical molecular weight, whereas no such bands were detected in the extracts of wild-type spores (Fig. 2). These data indicated the presence of the CotC-TreS and CotG-TreS fusion proteins in the spore coat.

**Fig. 3:**
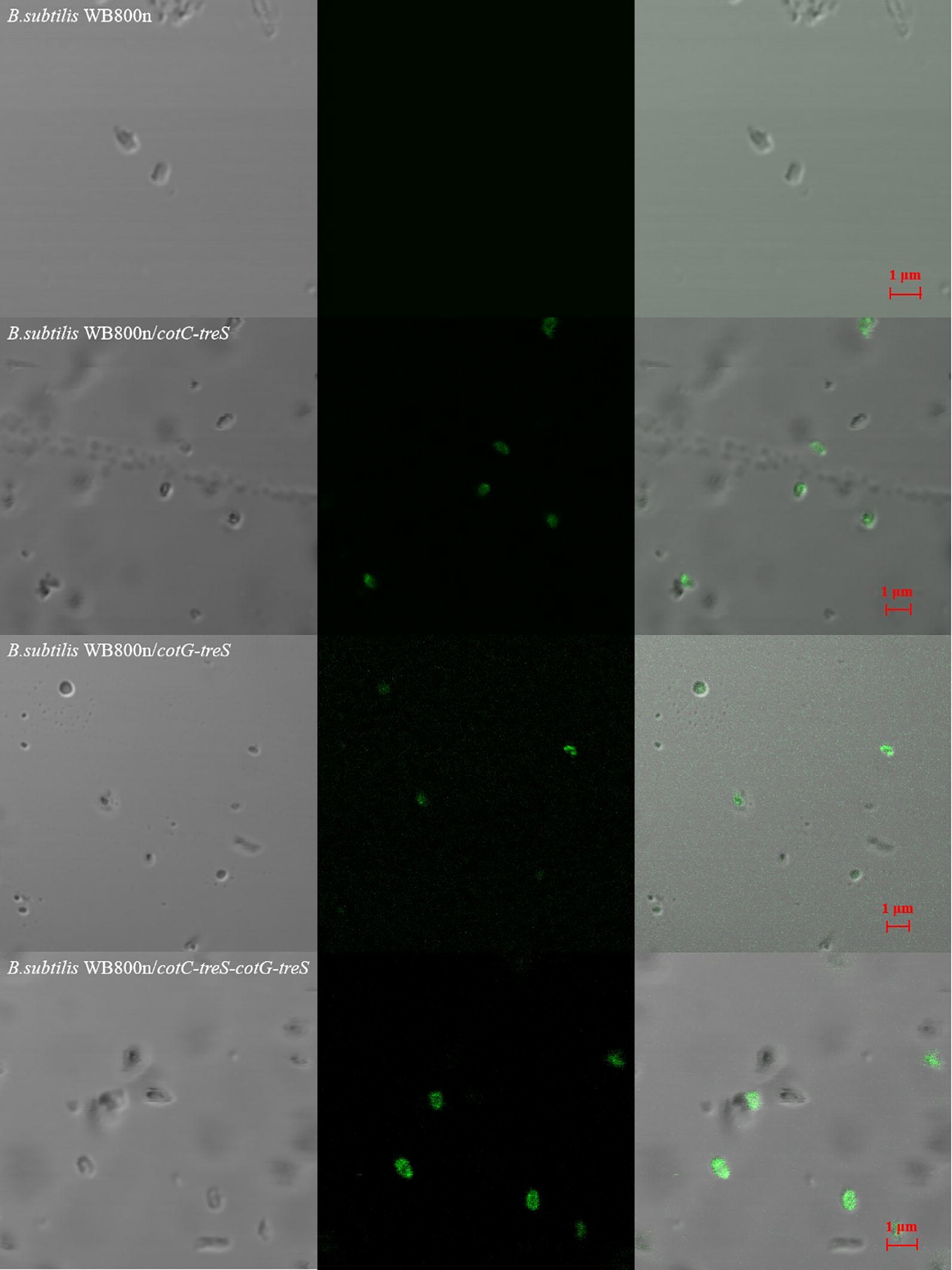
Localization of CotG-TreS, CotC-TreS, and CotC-TreS–CotG-TreS fusion proteins as assessed by immunofluorescence microscopy. Spores were incubated with the mouse anti-His tag antibody (1:60, primary antibody), followed by probing with the FITC-conjugated goat anti-rabbit IgG antibody (1:30, secondary antibody). The same exposure time was used for all immunofluorescence images. Photos from left to right correspond to white, fluorescent, and white-light superposition

To verify whether TreS was displayed on the spore surface, a fluorescence immunoassay was performed with the mouse anti-His-tag antibody (primary antibody) and a fluorescein isothiocyanate (FITC)-conjugated goat anti-rabbit IgG antibody (secondary antibody). Bright green fluorescence of different intensities was observed on the surface of the recombinant spores, but no fluorescence signal was detectable on the surface of control wild-type *B. subtilis* WB800n spores (Fig. 3), confirming that CotG-TreS, CotC-TreS, or CotC-TreS–CotG-TreS was present on the spore coat surface and was available for antibody binding, albeit with different binding efficiency.

To assess the amount of TreS anchored on the spore surface, the surface-displayed TreS was analyzed by a dot blot assay with the anti-His-tag antibody, with purified TreS containing the His-tag as a standard. The intensity of the various spots was quantified by densitometric analysis (Additional file 1). The number of TreS molecules displayed on the surface of a single spore was calculated next: 2.79 × 10^9^, and 1.38 × 10^9^, and 4.65 × 10^9^ for recombinant *B. subtilis* strains: WB800n/*cotC*-*treS*, and WB800n/*cotG*-*treS*, and WB800n/*cotC*-*treS*–*cotG*-*treS*, respectively.

### Measurements of TreS activity

**Fig. 4:**
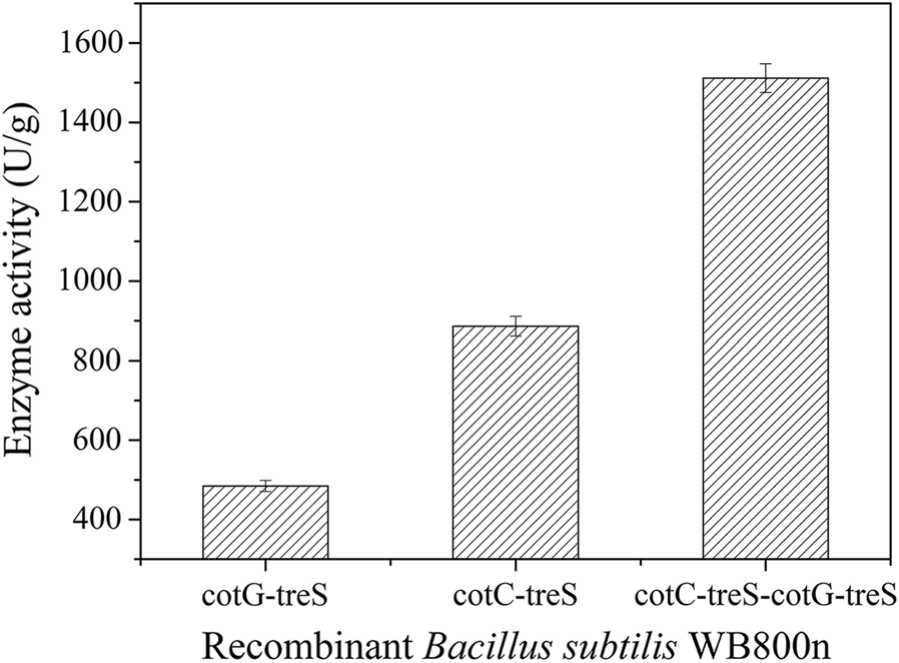
The TreS activity of spores respectively displaying CotC-TreS, CotG-TreS and CotC-TreS–CotG-TreS. The activity of the spore-displayed TreS was determined by measuring the formed d-trehalose in the reaction system composed of 300 g/L maltose, pH 8.0 PBS, and a certain amount of spore suspension in a total volume of 10 mL. d-Trehalose was quantified by HPLC. One unit of TreS activity was defined as the amount of enzyme catalyzing the formation of 1 μmol of d-trehalose per hour in the above reaction mixture

After proving that TreS was anchored on the surface of the spores, we analyzed the enzymatic activity by incubating purified spores with maltose (the substrate). The activity of the displayed TreS was determined by quantitation of the formed d-trehalose. The latter as a product of this reaction was quantified by high-performance liquid chromatography (HPLC), and its amount was found to be directly proportional to the amount of active TreS during the 2 h incubation. After 96 h of incubation with each recombinant strain in the TB medium, more than 90% of the bacteria formed spores. The spores and the remaining bacteria were collected by centrifugation. The TreS activity of the spores displaying CotC-TreS or CotG-TreS was 886.11 ± 9.48 and 484.51 ± 9.26 U/g, respectively (mean ± SD; Fig. 4). The TreS activity of spores displaying both CotC-TreS and CotG-TreS simultaneously was 1511.6 ± 58.09 U/g (Fig. 4). The greater activity with the combination of CotC and CotG was consistent with the finding that the estimated number of TreS molecules displayed on the surface per spore was greater when both CotC and CotG served as the anchor.

### *Characteristics of B. subtilis WB800n*(ΔsleB, ΔcwlJ)*/*cotC-treS–cotG-treS

To ensure spore stability and to prevent spore germination in the trehalose preparation system, genes *selB* and *cwlJ*, coding for germination-specific lytic enzymes, were deleted from the *B. subtilis* WB800n genome. *B. subtilis* WB800n(*ΔsleB*, *ΔcwlJ*)/*cotC*-*treS*–*cotG*-*treS* was inoculated into the medium for 96 h incubation at 37 °C. Cell numbers, spore numbers, the sporulation rate, and enzymatic activity were analyzed. The growth and spore formation were not affected in the recombinant bacteria with the deletion of genes *sleB* and *cwlJ* (Table 1).

**Table 1:** Comparison of recombinant *Bacillus subtilis* WB800N/cotC-treS–cotG-treS and *Bacillus subtilis* WB800N(*ΔsleB, ΔcwlJ*)/cotC-treS–cotG-treS ($\overline{\text{X}}$ ± S, n = 5)

Recombinant strain	Bacterial number (10^9^/mL)	Spore number (10^9^/mL)	Spore yield (%)	Enzyme activity of unit spore dry weight (U/g)
*Bacillus subtilis* WB800N/cotC-treS–cotG-treS	1.73 ± 0.065	1.56 ± 0.054	90.17 ± 3.3	2489 ± 119
*Bacillus subtilis* WB800N(*ΔsleB, ΔcwlJ*)/cotC-treS–cotG-treS	1.69 ± 0.074	1.52 ± 0.052	89.94 ± 4.1	2505 ± 121

Data presented are representative of five biological replicates (± SD)

**Fig. 5:**
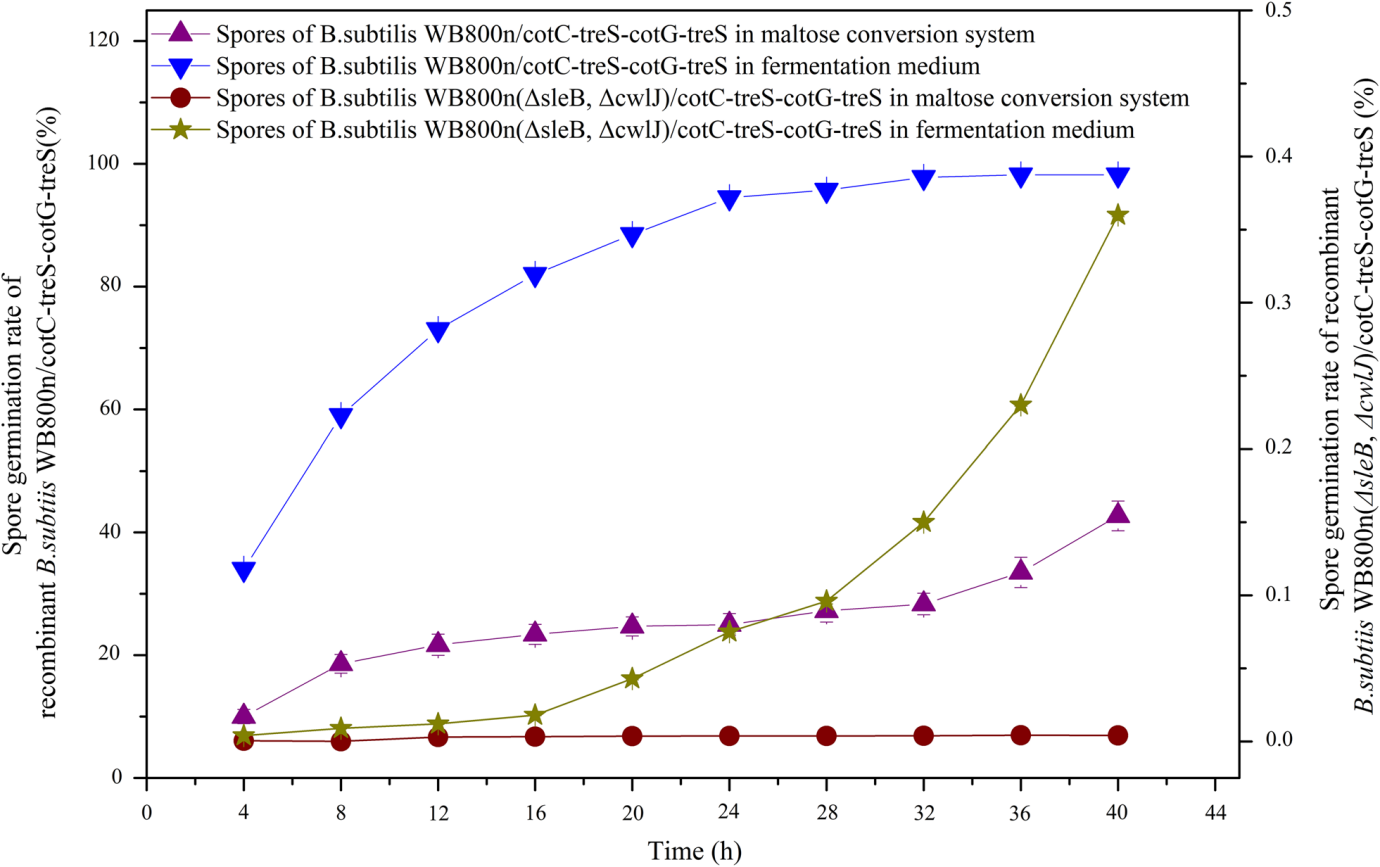
A comparison of germination rates of *B. subtilis* WB800n/*cotC*-*treS*–*cotG*-*treS* with and without *sleB* and *cwlJ* genes. Spores were added to the maltose (300 g/L) reaction solution, and the number of colonies was determined by the plate count method at 25 °C and pH 8.0 during 40 h incubation. Similarly, a defined number of spores were added to the fermentation medium to analyze their germination rate

Spores of *B. subtilis* WB800n(*ΔsleB*, *ΔcwlJ*)/*cotC*-*treS*–*cotG*-*treS* and *B. subtilis* WB800n/*cotC*-*treS*–*cotG*-*treS* were added to a maltose (300 g/L) reaction solution, and the number of germinated spores was determined by the plate count method at 25 °C and pH 8.0 during 40 h incubation. The samples were heated at 80 °C for 10 min every 4 h. Similarly, a defined number of spores were added to the fermentation medium to analyze their germination rate. The germination rate of the original strain in the maltose conversion system increased gradually with time and reached 20% at 12 h and 40% at 40 h (Fig. 5). In the fermentation medium, the germination rate reached 70% at 12 h and 90% at 20 h, suggesting that the original strain was easy to germinate in the medium and conversion system. By contrast, the germination rate of spores produced by *B. subtilis* WB800n(*ΔsleB*, *ΔcwlJ*)/*cotC*-*treS*–*cotG*-*treS* was less than 1% after 20 h and no germinating spores were evident in the maltose transformation system (Fig. 5). These findings indicated that the knockout of genes *sleB* and *cwlJ* effectively prevented the germination of spores during transformation.

### Enzymatic characterization of *B. subtilis* WB800n(ΔsleB, ΔcwlJ)/cotC-treS–cotG-treS spores

**Fig. 6:**
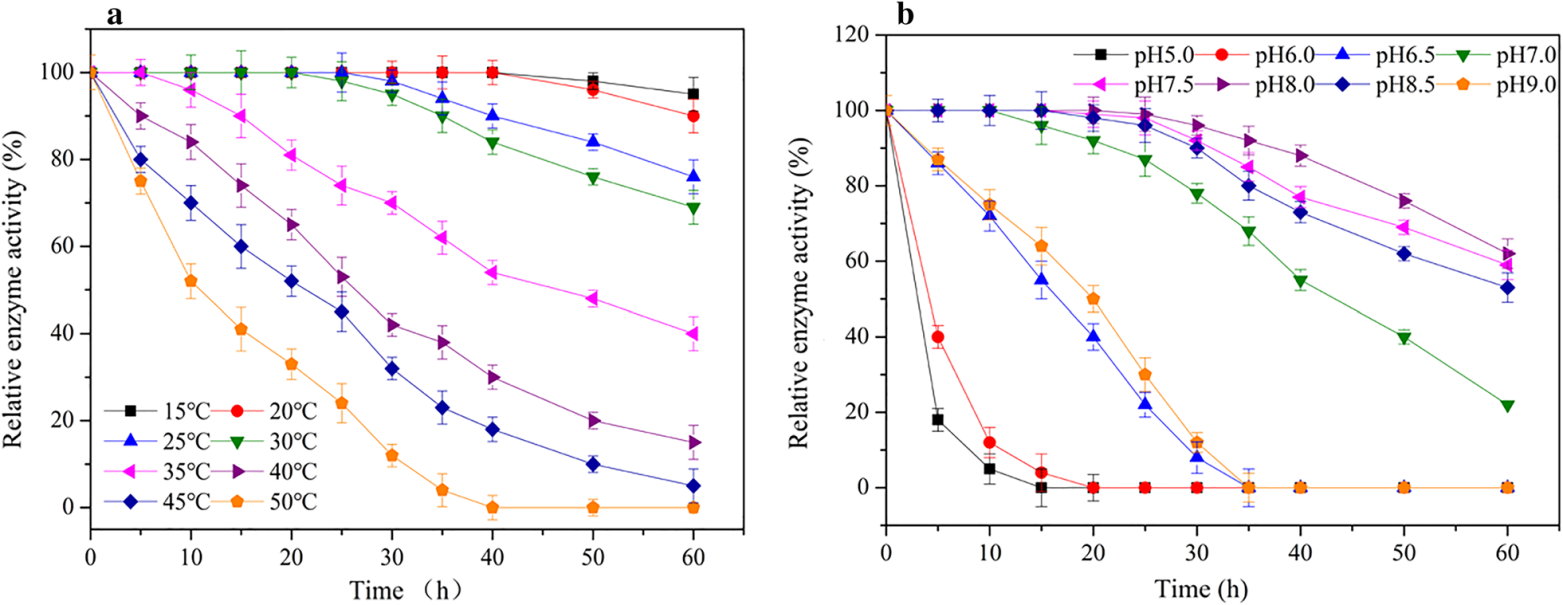
Relative enzymatic activity at different temperatures (15 °C, 20 °C, 25 °C, 30 °C, 35 °C, 40 °C, 45 °C, and 50 °C) and pH levels (5.0, 6.0, 6.5, 7.0, 7.5, 8.0, 8.5, and 9.0) at different time points. Residual enzymatic activity was determined at various time points by measurement of trehalose production via HPLC

The temperature found to be optimal for the activity of the displayed TreS was 25 °C (Additional file 2). Over 90% of the original activity remained at temperatures ranging from 10 to 40 °C (Additional file 2). The relative enzymatic activity of the displayed TreS exceeded 80% after incubation at 10–30 °C for 40 h, and more than 50% of the original activity remained after incubation at 50 °C for 10 h (Fig. 6a). These results indicated excellent thermal stability of the surface-localized TreS at 10–30 °C. Concerning pH, the surface-displayed TreS manifested maximum activity at pH 8.0, with 69.0% at pH 6.0 (Additional file 2). The relative activity exceeded 90% at pH ranging between 7.0 and 8.5 (Additional file 2). Next, pH stability of the displayed TreS was also tested at three pH levels. The relative activity was more than 98% after incubation at pH 8.0 for 25 h and exceeded 90% after incubation at pH 7.0 to 8.5 for 25 h (Fig. 6b).

### Production of trehalose by *B. subtilis* WB800n(ΔsleB, ΔcwlJ)/cotC-treS–cotG-treS on the spore surface

**Fig. 7:**
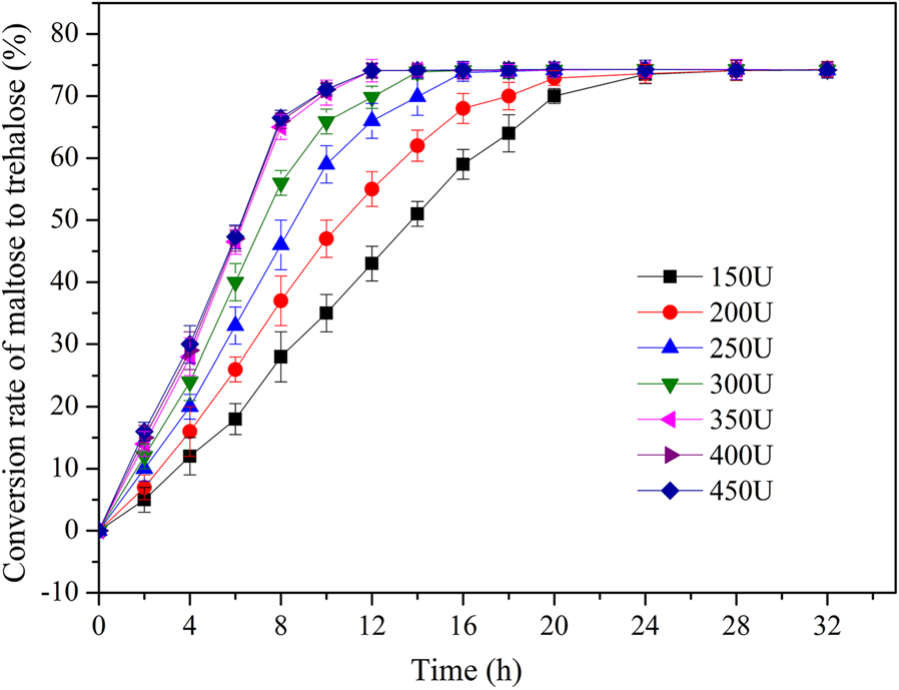
Conversion rate curves of trehalose at different spore-displayed treS amounts (i.e., activity ratios: 150, 200, 250, 300, 350, 400, or 450 U/[g maltose]) at 300 g/L maltose, pH 8.0, and 25 °C. The treS activity was adjusted for different numbers/concentrations of spores

To enhance the efficiency of trehalose production, the activity of spore-displayed treS during biotransformation was evaluated in a 2000 mL shake flask with 500 mL of a reaction solution containing 150 g of d-maltose. The TreS activity was adjusted for different numbers/concentrations of spores, thus resulting in 150–450 U/(g maltose). Trehalose production kept rising with time, whereas the conversion rate finally reached an equilibrium, and the highest conversion rate of maltose to trehalose was 74.1% (Fig. 7). The highest conversion rate of maltose to trehalose was observed at 350 U/(g maltose) during the 12 h biotransformation. At an enzymatic activity of 400–450 U/g maltose, the conversion rate and conversion time were not significantly better (Fig. 7).

**Fig. 8:**
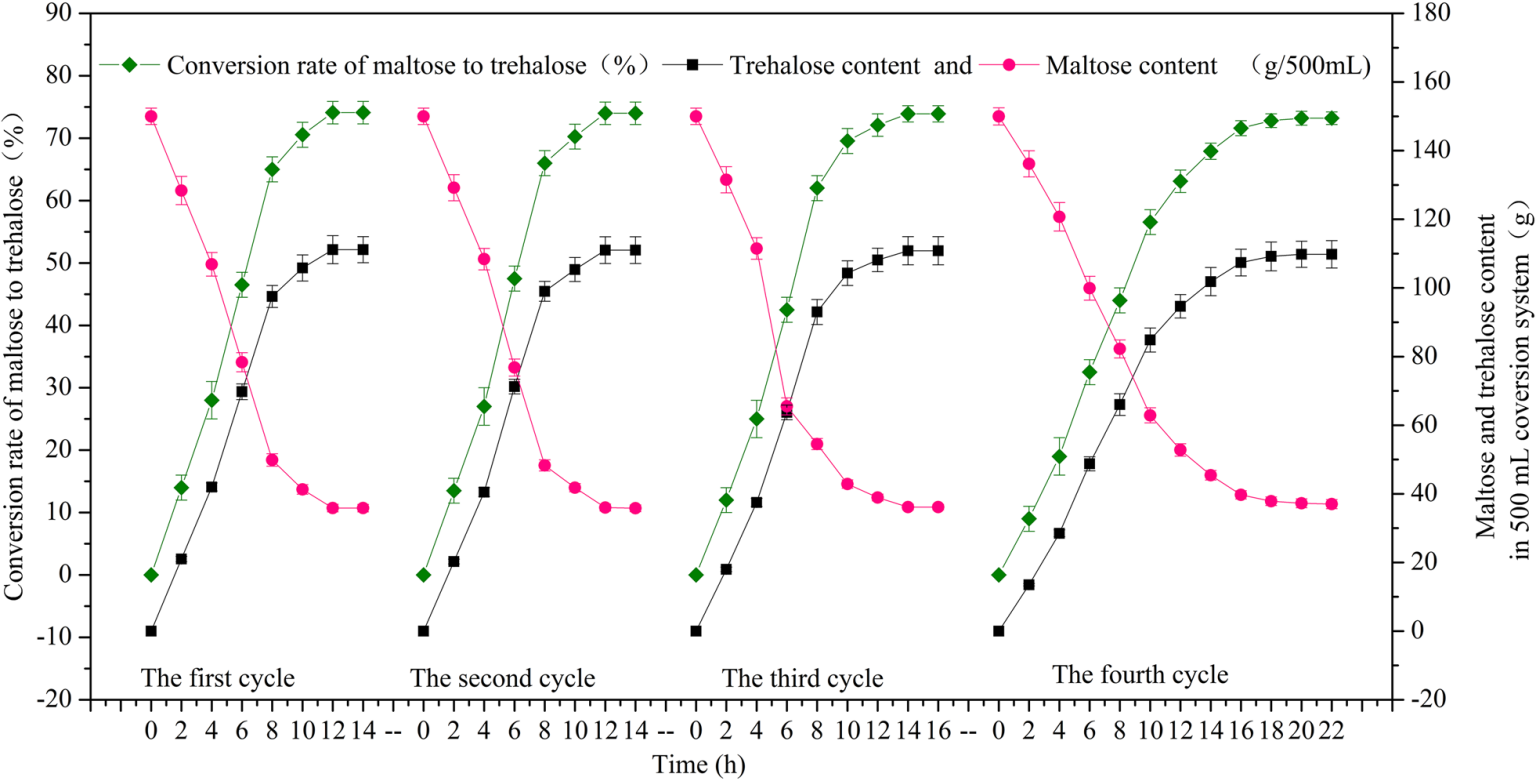
The change curve of the conversion rate, trehalose production, and maltose consumption evaluated in a 2000 mL shake flask containing 500 mL of the reaction solution in four repeated biotransformation cycles by means of the spores of *B. subtilis* WB800n(*ΔsleB*, *ΔcwlJ*)/*cotC*-*treS*–*cotG*-*treS*

Considering the production efficiency and economic efficiency, activity ratio 350 U/(g d-maltose) was finally chosen for the biotransformation. To improve the application efficiency of the surface-displayed TreS, d-trehalose production was evaluated in a 2000 mL shake flask containing 500 mL of the reaction solution under the optimized conditions. Four cycles were carried out and afforded a conversion rate greater than 73.0% (Fig. 8). The profiles of d-maltose production and d-maltose consumption in the repeated biotransformation reactions involving the recombinant spores are presented in Fig. 8. At the end of each cycle, d-trehalose production reached 109.8 to 111.15 g in the 500 mL reaction solution. In the first cycle, the conversion rate of d-maltose to d-trehalose was 74.1%, and the maximum production of d-trehalose was 111.15 g in the 500 mL reaction solution at 12 h, with 18.525 g/(L h) d-trehalose volumetric productivity. In the second cycle, the conversion rate reached 74.0%, and the production of d-trehalose was 111.0 g in the 500 mL reaction solution at 12 h, with d-trehalose volumetric productivity of 18.5 g/(L h). In the third cycle, the conversion rate reached 73.9%, and the production of d-trehalose was 110.85 g in the 500 mL reaction solution at 14 h, amounting to d-trehalose volumetric productivity of 15.836 g/(L h). In the fourth cycle, the conversion rate reached 73.2%, and the production of d-trehalose was 109.8 g in 500 mL reaction solution at 22 h, meaning d-trehalose volumetric productivity of 9.98 g/(L h). These results meant that the conversion rate and d-trehalose volumetric productivity decreased after the third cycle of conversion. Therefore, it was more economical to carry out four cycles of conversion.

## Discussion

*Bacillus subtilis* spores have been used most commonly for the surface display of various food-related or human antigens or enzymes owing to these spores’ excellent stability, low cost, safety, ease of preparation, and resistance to harsh conditions [32].

Successful surface display of a target protein requires selection of appropriate anchoring proteins that contain signal sequences that effectively promote the transport of the target protein to the cell surface and this protein’s immobilization and stability there [33]. *B. subtilis* spores contain a variety of spore coat proteins, but the preferred anchoring proteins are the outer-coat proteins [32]. CotB, CotG, and CotC are the most popular outer-coat proteins [22, 23, 26, 29]. In our study, CotC and CotG were chosen to display TreS on the *B. subtilis* spore surface. TreS was successfully displayed on the spore surface when fused to CotC, CotG, or both. TreS fused with the CotC–CotG combination was more effectively localized to the spore surface and manifested greater activity in comparison with the fusion to either CotC or CotG alone, as verified by western blotting, immunofluorescence, dot blot, and enzymatic-activity assays. Another study has shown that the same passenger protein might yield varied results when displayed with different coat proteins [23], in agreement with the results of the present study. Of note, the enzymatic activity per unit dry spore weight of TreS fused with CotC–CotG (1511.6 U/g) and the number of molecules on the spore surface (4.65 × 10^9^) exceeded the respective values when the anchoring protein was CotG alone (484.51 U/g and 1.38 × 10^9^) or CotC alone (886.6 U/g and 2.79 × 10^9^). These findings have not been reported in other studies on TreS. Our data suggest that the quantity of TreS displayed on the surface of a spore can be increased in some ways. This finding may be beneficial for the reduction of unit preparation costs and for improvement of production efficiency. One study suggests that genes *cotA*, *cotB*, and *cotC* are coordinately expressed as aggregation nears completion [34]. Our study revealed that CotC and CotG can act synergistically, and the reason needs further research.

*Bacillus subtilis* spores are extremely stable in their dormant state. Nonetheless, germination does occur under the influence of factors that include cationic surfactants, high pressure, and specific nutrients. A crucial event in the process of spore germination is degradation of a thick peptidoglycan-containing layer termed the cortex. Hydrolysis of the cortex allows the spore core to expand and hydrate to levels found in vegetative cells. If the degradation of cortex peptidoglycan is blocked, then spore viability can be drastically reduced because spore metabolism and macromolecule synthesis cannot begin [35–37]. There are two redundant cortex-lytic enzymes in the spores of *Bacillus* species: *CwlJ* and *SleB*. Both are specific for peptidoglycan. Double mutant spores that are deficient in both have negligible viability [31, 38–40]. In our study, to avoid the germination of spores in the conversion system containing maltose, phosphate buffer, and surface-displaying spores, genes *sleB* and *cwlJ* were knocked out in recombinant *B. subtilis* WB800n. This double gene knockout did not affect sporulation of recombinant *B. subtilis* WB800n, but spore germination was greatly inhibited in the conversion system, thereby increasing spore stability.

Display of proteins on the spore surface may lead to changes in protein structure and surface charge, thus increasing relative activity, thermal stability, pH stability, and reusability of an enzyme. In our study, the spore surface-displayed mutant TreS was active within a limited temperature range 10 to 60 °C, with an optimal temperature of 25 °C, as in another report, which described the optimal temperature *of Pseudomonas putida* wild-type TreS displayed on the *Pichia pastoris* cell surface was also 25 °C [41]. More than 50% of spore surface-displayed mutant TreS activity turned out to be retained at pH ranging from 6.5 to 9.5, with the optimum pH of 8.0. The pH profile of mutant TreS display on the spore surface, at the optimum pH of 8.0, has been previously reported for wild-type TreS displayed on the *Pichia Pastoris* cell surface and expressed in *E. coli* [41, 42]. In our conversion system, the mutant TreS displayed on the surface of spores could be recycled four times at 25 °C, pH 8.0, and 300 g/L maltose, with the conversion rate of 74.1% in the first cycle and 73.0% for 16 h in the fourth cycle. Another study has revealed that wild-type TreS displayed on the *Pichia Pastoris* cell surface can be recycled three times, with a conversion rate of 64.0% in the first cycle and 63.0% in the third cycle [41]. This difference in conversion rate and reusability times may be caused by mutation or different expression systems [43]. It appears that mutant TreS displayed on the surface of *B. subtilis* spores has no effect on thermal stability and pH stability but improves reusability of the enzyme.

## Conclusion

The spore surface display system based on food grade *B. subtilis* with CotC and CotG as the carrier seems to be a powerful technology for TreS expression, with good reusability and a 73.0% conversion rate after four cycles. The TreS surface display system constructed in this study offers a promising option for trehalose production.

## Methods

### Bacterial strains and media

The bacteria strains, plasmids, and primers used in this study are listed in Additional file 3. *B. subtilis* WB800n was employed for the spore surface display and amplification of genes *cotC* and *cotG*. *E. coli* DH5α was used to clone the recombinant plasmid. Plasmid pDG1730 served as the *E. coli*–*B. subtilis* shuttle vector.

#### Recombinant-plasmid construction

Amplification of *cotC* and *cotG* from the *B. subtilis* WB800n chromosome was conducted with primer pair cotC-F/cotC-R or cotG-F/cotG-R, respectively. DNA harboring the mutant V407M/K490L/R680E *treS* gene from *P. putida* ATCC47054 was amplified by PCR from pET15b-treS as the template with primer pair cotC-treS-F-1/treS-R or cotG-treS-F-1/treS-R, respectively. To display TreS on the surface of *B. subtilis* WB800n spores, two fusion genes (*cotC*-*treS* and *cotG*-*treS*) were constructed by the multifragment seamless cloning technology [44]. *Bam*HI-and-*Hin*dIII-digested *cotC*-*treS* and *cotG*-*treS* PCR products were ligated at the corresponding sites into spore surface display vector pDG1730 [45] to obtain the respective recombinant plasmids pDG1730-CotC-treS and pDG1730-CotG-treS, in which CotC-treS and CotG-treS are coexpressed under the control of the *cotC* promoter and *cotG* promoter, respectively. The recombinant plasmids were transfected into *E. coli* DH5α competent cells, and colony PCR was performed to verify whether the desired genetic construct was present. The *cotC*-*treS* and *cotG*-*treS* genes were amplified from pDG1730-cotC-treS and pDG1730-cotG-treS, respectively, as the template with primer pair cotC-treS-F-2/cotC-treS-R-2 or cotG-treS-F-2/cotG-treS-R-2, respectively. Next, another fusion gene, *cotC*-*treS*–*cotG*-*treS*, was constructed by the multifragment seamless cloning technology using Exnase MultiS (Vazyme Biotech, China). The *Bam*HI-and-*Hin*dIII-digested *cotC*-*treS*–*cotG*-*treS* PCR product was ligated at the corresponding sites into spore surface display vector pDG1730 to obtain recombinant plasmid pDG1730-CotC-treS–CotG-treS. The recombinant plasmid was transfected into *E. coli* DH5α competent cells, and colony PCR was performed to verify whether the desired genetic construct was present. The construction process is depicted in Additional files 4 and 5.

#### Construction of recombinant *B. subtilis* WB800n and chromosomal integration

Recombinant plasmids pDG1730-CotC-treS, pDG1730-CotG-treS, and pDG1730-CotC-treS–CotG-treS extracted from the respective recombinant *E. coli* DH5α clones were electroporated into competent *B. subtilis* WB800n cells, to integrate gene *cotC*-*treS*, *cotG*-*treS*, or *cotC*-*treS*–*cotG*-*treS* into the *B. subtilis* WB800n chromosome at the *amyE* locus by double cross-over recombination. The transformed cells were incubated in the LB medium and cultured at 37 °C overnight with vigorous shaking (200 rpm), followed by seeding onto an LB plate containing 100 μg/ml spectinomycin and incubation at 37 °C for 16 h. Colonies resistant to spectinomycin were obtained, and colonies with *cotC*-*treS*, *cotG*-*treS*, or *cotC*-*treS*–*cotG*-*treS* integrated at the *B. subtilis amyE* locus were identified by assaying amylase activity. The integration of *cotC*-*treS*, *cotG*-*treS*, or *cotC*-*treS*–*cotG*-*treS* at the *amyE* locus was expected to yield an amylase-negative phenotype on LB plates containing 1% starch. After incubation at 37 °C for 16 h, the plates were stained with iodine to examine the amylase activity. The blue color was produced by the starch–iodine reaction in the presence of fusion gene *cotC*-*treS*, *cotG*-*treS*, or *cotC*-*treS*–*cotG*-*treS* in the *B. subtilis* chromosome. No blue color was observed in the *B. subtilis* WB800n control when iodine was added because the expression of *amyE* resulted in hydrolysis of the starch in the plate. Integration-positive clones were confirmed by PCR. The strategy for the chromosomal integration of the *Cot*-*treS* gene fusions is illustrated in Additional file 6.

### Spore preparation

To obtain spores with TreS displayed on the surface, recombinant *B. subtilis* WB800n was cultivated in the TB medium (12 g/L peptone, 24 g/L yeast extract, 4 mL/L glycerol, 72 mM K_2_HPO_4_, and 17 mM KH_2_PO_4_). After cultivation in TB at 37 °C and 200 rpm for 96 h, the spores and sporangial cells of *B. subtilis* WB800n harboring the recombinant plasmids were harvested by centrifugation and resuspended in 100 mM sodium phosphate buffer (pH 7.5). The spore suspension was purified by a 0.5% lysozyme treatment at 37 °C for 1 h to dissolve residual sporangial cells and then centrifuged at 1000*g* for 15 min. The purified spores were washed sequentially with 1 M NaCl, 1 M KCl, and phosphate buffer and then resuspended at 4 °C in phosphate buffer free of phenylmethylsulfonyl fluoride. The spores were enumerated after incubation at 80 °C for 10 min to kill *B. subtilis* WB800n cells and were spread on plates.

### Western blotting and dot blot assays

The spores obtained as described above were washed and resuspended in SDS-DTT buffer (1% sodium dodecyl sulfate, 50 mM dithiothreitol, and 0.1 M NaCl), incubated at 37 °C in a water bath for 2 h, washed three times with 50 mmol/L Tris–HCl (pH 7.5), and finally resuspended in a pyrolysis solution (50 mmol/L Tris–HCl [pH 7.5] and 0.5 mol/L EDTA) in a centrifuge tube. The tube was placed on ice for ultrasonication (300 W in bursts of 2–4 s for 15 min). The solution was centrifuged at 2000*g* for 20 min at 4 °C. The spores were precipitated with 50 mmol/L Tris–HCl (pH 7.5). After spore coat protein was extracted from the purified spore suspension, the surface TreS was characterized by SDS-PAGE and western blotting. Briefly, the proteins were electrotransferred to a polyvinylidene difluoride membrane. The membrane was sealed with sealing liquid at room temperature for 1 h. Next, the membrane was incubated with the anti-His-tag antibody (1:3000, Proteintech) overnight at 4 °C and washed three times with Tris-buffered saline with 0.1% Tween 20 (5 min each wash), followed by incubation with a horseradish peroxidase-conjugated goat anti-mouse IgG antibody (H + L, 1:2000; ZSGB-BIO, China) at room temperature for 1 h and three more washes with Tris-buffered saline containing 0.1% Tween 20 (10 min each). The membrane was covered with Immu Plus ECL (BOSTER BIO, China) and examined via a gel imaging system (Bio-Rad, USA). Next, to confirm the display of TreS, the purified recombinant spores were treated with 0.1% trypsin, 0.1% bromelain, or 0.1% proteinase K in potassium phosphate buffer (0.05 M, pH 6.5) at 37 °C for 1 h. *B. subtilis* spores that were not treated with a protease served as the control. Serial dilutions of extracted proteins and purified TreS were subjected to a dot blot assay. Briefly, a series of dilutions of standard and test samples were prepared. Aliquots of the serial dilutions were placed on the membrane in a row, and the membrane was dried. The antibody incubation process was the same as described for the western blot, except for the primary antibody incubation, which lasted 1 h at room temperature. The filters were then visualized by staining with 5-bromo-4-chloro-3-indolyl-phosphate/nitro blue tetrazolium color development solution (Bio-Rad) and densitometric analysis on a Fluor-*S* Multimager (Bio-Rad). The average intensity of each sample region was analyzed in the ImageJ software. Formula 1 was used to convert the average densitometric value of each sample to optical density (OD). The total OD value of the sample was obtained by multiplying the OD by the area of the sample region. The total OD and dot quality of each standard sample were used to draw the standard curve, and the standard curve formula was determined. The total OD of each sample to be tested was substituted into the standard formula, and then the dot quality of each sample to be tested was obtained. The number of TreS molecules per spore was calculated according to Formula 2:

1 $$OD = \lg \frac{255}{255 - gray}$$

2 $$n_{0} = \frac{{mN_{A} }}{nM}$$

where *n*_*0*_ represents the number of molecules displayed on a spore, *m* is the total mass of TreS extracted from *n* spores, *N*_*A*_ is Avogadro’s constant, and *M* denotes the molecular weight of TreS.

### Immunofluorescence microscopy

Spores were resuspended in TE buffer (20 mM Tris–HCl [pH 7.5] and 10 mM EDTA) containing 2 mg/mL lysozyme. After a 3 min incubation, samples were washed three times with PBS (pH 7.4) before blocking with 3% skimmed milk in PBS for 30 min at room temperature and washing another three times with PBS. The samples were incubated for 2 h at 4 °C with a mouse anti-His-Tag antibody (1:60; Proteintech, USA), washed three times, and then incubated with FITC-conjugated AffiniPure Sheep Anti-Mouse IgG (H + L, 1:30; BOSTER BIO) for 2 h at 4 °C. Fluorescence was examined at an excitation wavelength of 488 nm by confocal laser scanning microscopy (Leica, Germany).

### An activity assay of TreS displayed on the surface of spores

The recombinant bacteria were grown at 37 °C on LB agar plates containing 50 mg/mL spectinomycin. Single colonies were incubated in 10 mL of the medium supplemented with 50 mg/mL spectinomycin and cultured for 12 h and then transferred into 100 mL of the TB medium for 96 h fermentation at 200 rpm and 37 °C. After that, 10 mL of the fermentation liquid was centrifuged to measure the dry weight of the bacteria, and 1 mL of the fermentation liquid was subjected to a spore count. The remaining fermentation liquid was centrifuged and the precipitate was resuspended in pH 8.0 PBS, followed by ultrasonic disruption (power 300W, working 5 s, interval 10 s) for 15 min. Then, the spore suspension was purified by 0.5% lysozyme treatment at 37 °C for 1 h to dissolve residual sporangial cells and then centrifuged at 1000*g* for 15 min, followed by washing steps in 1 M NaCl, 1 M KCl, and phosphate buffer in that order. The activity of the spore-displayed TreS was determined by measuring the formed d-trehalose in the reaction system composed of 300 g/L maltose, pH 8.0 PBS, and a certain amount of spore suspension in a total volume of 10 mL [37]. After incubation at 25 °C for 60 min, the reaction was terminated by immersion of the reaction tubes in boiling water for 10 min [37]. After chilling, d-trehalose was quantified by HPLC (Shimadzu-GL Sciences, Japan). The apparatus was equipped with an inertsil-NH_2_ column (4.6 × 250 mm, Shimadzu) operating at 40 °C. Separation was achieved by pumping acetonitrile: water (75:25, v/v) at a flow rate of 1.0 mL/min for 20 min. One unit of TreS activity was defined as the amount of enzyme catalyzing the formation of 1 μmol of d-trehalose per hour in the above reaction mixture. The experiments were performed three times, with five independent biological replicates each time.

### The knockout of *B. subtilis* WB800n genes selB and cwlJ

To ensure spore stability and avoid spore germination in the trehalose preparation system, genes *selB* and *cwlJ* were deleted from the *B. subtilis* WB800n genome. The upstream and downstream homologous arms of *selB* and *cwlJ* were amplified with primer pairs sleB-F/sleB-R and cwlJ-F/cwlJ-R, respectively, and the *B. subtilis* 168 genome as a template. The kanamycin resistance gene was amplified from the pPIC9k plasmid as a template and Km-F/Km-R as the primer pair. The *selB* and *Kan* genes were fused by means of selB-F/Km-R as the primer pair by splicing overlap extension PCR, followed by electroporation of the *selB*-*Kan* fusion gene into *B. subtilis* W800N. The electrotransformed bacteria were plated on LB agar supplemented with kanamycin (25 μg/mL) and grown at 37 °C for 16 h to select *selB* gene-deficient cells. Knockout-positive clones verified by colony PCR were designated *B. subtilis* WB800N(*ΔselB*). Then, using the pPIZαA plasmid as the template and the zeo-F/zeo-R primer pair, the zeomycin resistance gene (*Zeo*) was amplified, followed by fusion with the *cwlJ* gene using cwlJ-F/Zeo-R as the primer pair by splicing overlap extension PCR. The *cwlJ*-*Zeo* fusion gene was electroporated into *B. subtilis* W800N(*ΔselB*) and the bacteria were plated on LB agar supplemented with zeomycin (100 μg/mL) to select a *cwlJ* gene-deficient stain, which was designated as *B. subtilis* WB800n(*ΔselB,ΔcwlJ*). The construction of the latter strain is detailed in Additional file 7.

### Characterization of *B. subtilis* WB800n(ΔsleB, ΔcwlJ)/cotC-treS–cotG-treS

*Bacillus subtilis* WB800n(*ΔsleB*, *ΔcwlJ*)/*cotC*-*treS*–*cotG*-*treS* was inoculated into the medium consisting of 20 g/L glucose, 12 g/L tryptone, 24 g/L yeast extract, 72 mM K_2_HPO_4_, 17 mM KH_2_PO_4_, and 2.5 g/L MgCl_2_ for 96 h incubation at 37 °C. The bacterial number, spore numbers, spore yield, and enzymatic activity was analyzed.

The effect of temperature on TreS displayed on the spore surface was examined in a standard activity assay conducted at various temperatures ranging from 10 to 65 °C. To test TreS thermal stability, the displayed TreS was incubated at 15 °C, 20 °C, 25 °C, 30 °C, 35 °C, 40 °C, 45 °C, or 50 °C and residual enzymatic activity was determined at various time points via measurement of trehalose production by HPLC. The effect of pH on the displayed TreS was determined using 100 mM potassium phosphate buffer at pH ranging from 4.0 to 10.0. To determine pH stability, the displayed TreS was incubated at 4 °C in a buffer with pH of 5.0, 6.0, 6.5, 7.0, 7.5, 8.0, 8.5, or 9.0. Residual enzymatic activity was determined as a function of time at the optimal temperature and pH of 25 °C and 8.0, respectively. The conversion rate of maltose to trehalose (%) was calculated as trehalose content in the conversion system (wt, g) divided by the initial total maltose content (wt, g). The experiments were conducted three times, with five independent biological replicates each time.

### Production of trehalose using *B. subtilis* WB800n(ΔsleB, ΔcwlJ)/cotC-treS–cotG-treS displayed on the spore surface

To optimize the biotransformation conditions, 500 mL reaction mixtures composed of 150 g of d-maltose in 10 mM phosphate buffer (pH 8.0) and different amounts (in terms of activity) of the surface-displayed TreS were prepared in 2000 mL shake flasks. The chosen activity ratios were 150, 200, 250, 300, 350, 400, and 450 U/(g maltose). The measurements were carried out via reaction cycles at the optimal temperature and pH of 25 °C and 8.0, respectively, at 60 rpm. After each cycle, the reaction solution was centrifuged at 2000*g* for 10 min, and the recovered material (pellet) was resuspended in 10 mM potassium phosphate buffer (pH 8.0) for the next cycle. The bioconversion of maltose to trehalose was analyzed by HPLC. The conversion rate of maltose to trehalose (%) was computed as trehalose content in the conversion system (wt, g) divided by the initial total maltose content (wt, g). The experiments were conducted three times, with five independent biological replicates each time.

## Additional files

**Additional file 1.** The dot blot assay of TreS displayed on spore surface from different recombinant bacteria. 1: TreS standard; C+G: TreS displayed on the spore surface of *B. subtilis* WB800n/*cotC*-*treS*–*cotG*-*treS*; C: TreS displayed on the spore surface of *B. subtilis* WB800n/*cotC*-*treS*; G: TreS displayed on the spore surface of *B. subtilis* WB800n/cotG-treS.

**Additional file 2.** Optimal pH, temperature, and tolerance of TreS displayed on the spore surface.

**Additional file 3.** The bacterial strains, plasmids, and primers used in this study.

**Additional file 4.** Construction of recombinant plasmids pDG1730-CotC-treS and pDG1730-CotG-treS.

**Additional file 5.** Construction of recombinant plasmid pDG1730-CotC-treS–CotG-treS.

**Additional file 6.** The strategy for the chromosomal integration of the *Cot*-*treS* fusion genes.

**Additional file 7.** Construction of recombinant *B. subtilis* WB800n with deletion of genes *sleB* and *cwlJ*.

## Data Availability

All data generated or analysed during this study are included in this published article and its additional files.

## Abbreviations

FITC: fluorescein isothiocyanate
HPLC: high-performance liquid chromatography
LB: Luria–Bertani
OD: optical density
TreS: trehalose synthase
