# Supplementary material for: Production of trehalose with trehalose synthase expressed and displayed on the surface of Bacillus subtilis spores
Source: Microb Cell Fact. 2019 Jun 3;18:100. doi: 10.1186/s12934-019-1152-7 (PMC6547511; doi:10.1186/s12934-019-1152-7)
Supplement: Supplementary file 1 — Additional file 1. The dot blot assay of TreS displayed on spore surface from different recombinant bacteria. 1: TreS standard; C+G: TreS displayed on the spore surface of B. subtilis WB800n/cotC-treS–cotG-treS; C: TreS displayed on the spore surface of B. subtilis WB800n/cotC-treS; G: TreS displayed on the spore surface of B. subtilis WB800n/cotG-treS. [file 12934_2019_1152_MOESM1_ESM.docx]

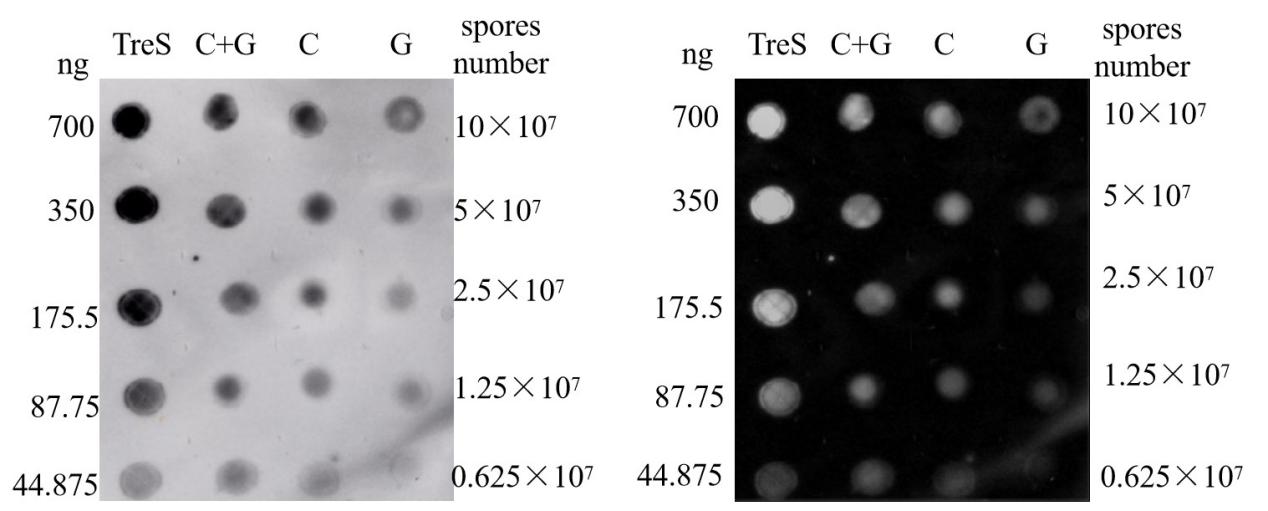


Additional file 1. Dot-blot analysis of TreS displayed on spore surface from different recombinant bacteria. 1: TreS standard; C+G: TreS displayed on spores surface of recombinant *B. subtilis* WB800n/cotC-treS-cotG-treS; C: TreS displayed on spores surface of recombinant *B. subtilis* WB800n/cotC-treS; G: TreS displayed on spores surface of recombinant *B. subtilis* WB800n/cotG-treS.
