# Supplementary figures and images for: Production of trehalose with trehalose synthase expressed and displayed on the surface of Bacillus subtilis spores
Source: Microb Cell Fact. 2019 Jun 3;18:100. doi: 10.1186/s12934-019-1152-7 (PMC6547511; doi:10.1186/s12934-019-1152-7)

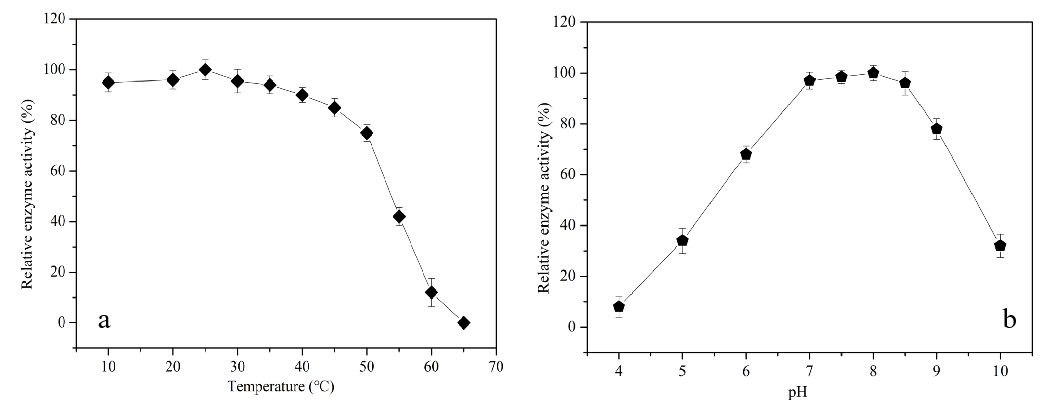


Additional file 2. Optimal pH, temperature, and tolerance of TreS displayed on the spore surface.

Supplement: Supplementary file 2 — Additional file 2. Optimal pH, temperature, and tolerance of TreS displayed on the spore surface. [file 12934_2019_1152_MOESM2_ESM.docx]

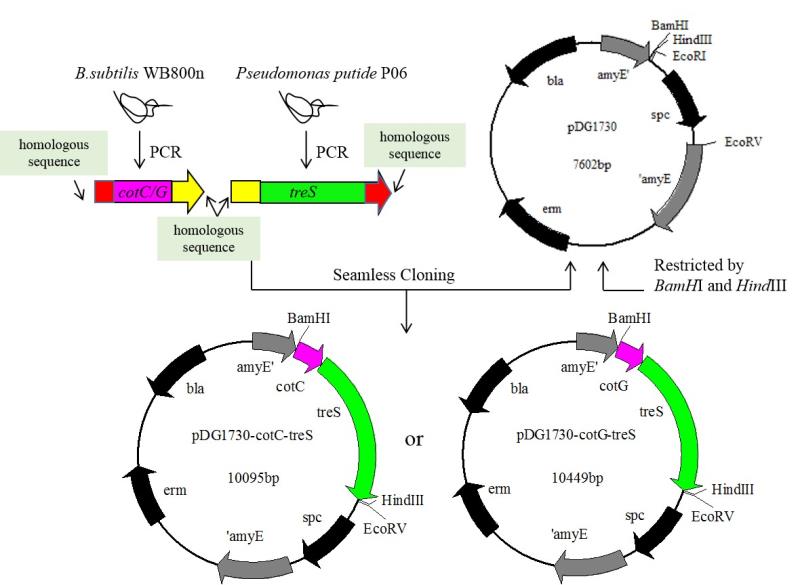


Additional file 4. Construction of recombinant plasmids pDG1730-CotC-treS and pDG1730-CotG-treS.

Supplement: Supplementary file 4 — Additional file 4. Construction of recombinant plasmids pDG1730-CotC-treS and pDG1730-CotG-treS. [file 12934_2019_1152_MOESM4_ESM.docx]

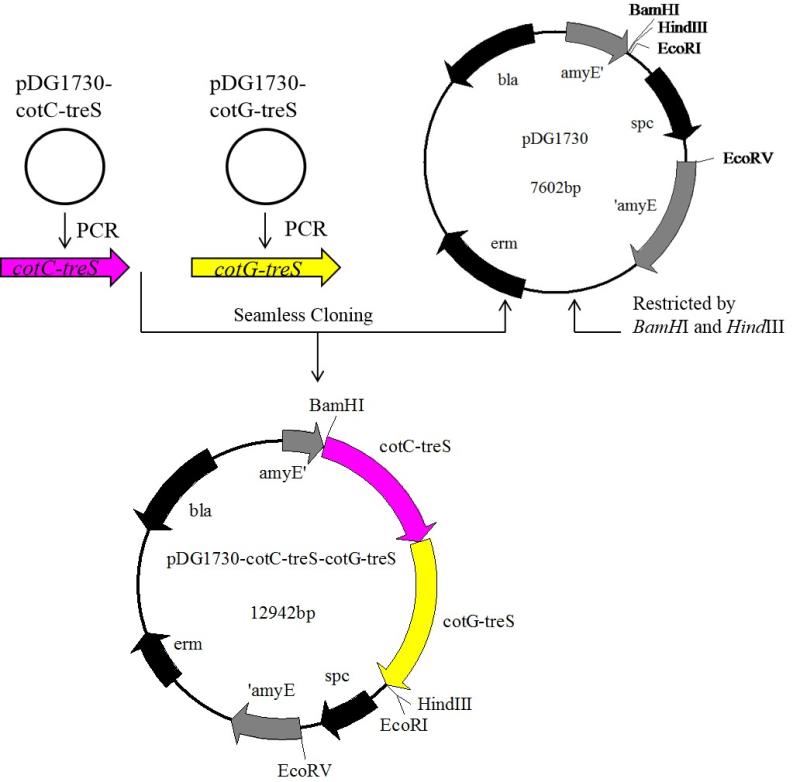


Additional file 5. Construction of recombinant plasmid pDG1730-CotC-treS -CotG-treS.

Supplement: Supplementary file 5 — Additional file 5. Construction of recombinant plasmid pDG1730-CotC-treS–CotG-treS. [file 12934_2019_1152_MOESM5_ESM.docx]

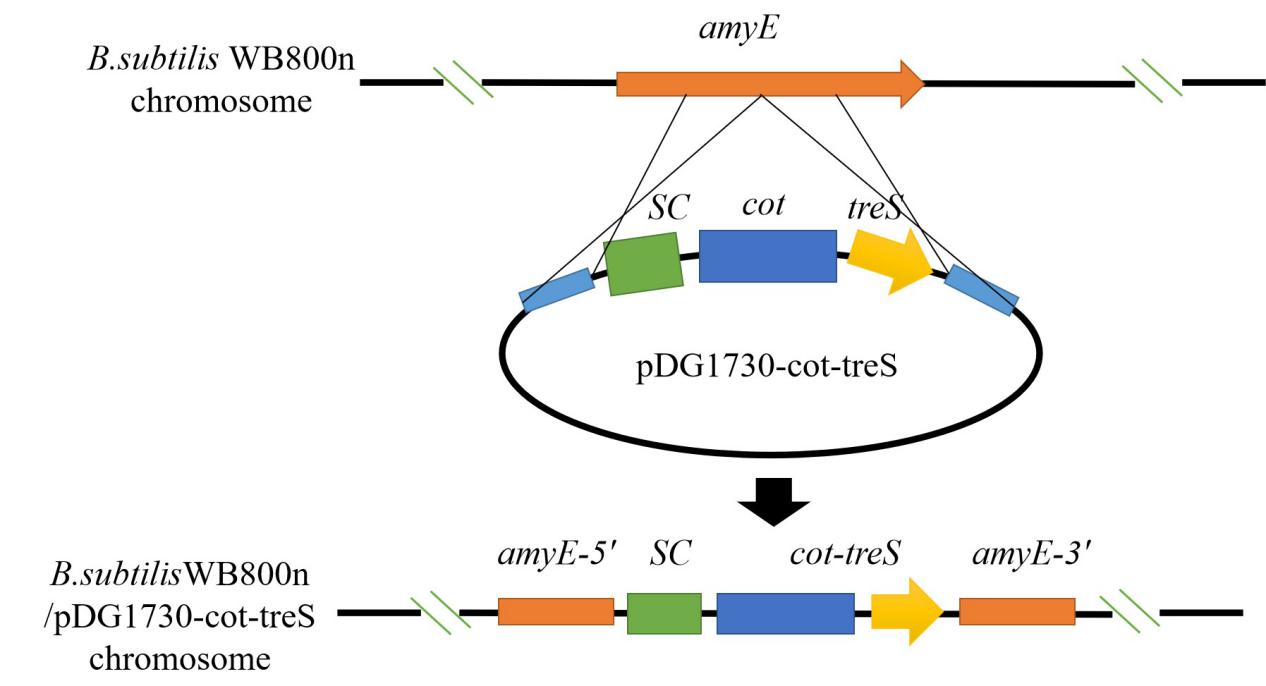


Additional file 6 Strategy for the chromosomal integration of the Cot-treS gene fusions

Supplement: Supplementary file 6 — Additional file 6. The strategy for the chromosomal integration of the Cot-treS fusion genes. [file 12934_2019_1152_MOESM6_ESM.docx]
