# Supplementary material for: Production of trehalose with trehalose synthase expressed and displayed on the surface of Bacillus subtilis spores
Source: Microb Cell Fact. 2019 Jun 3;18:100. doi: 10.1186/s12934-019-1152-7 (PMC6547511; doi:10.1186/s12934-019-1152-7)
Supplement: Supplementary file 3 — Additional file 3. The bacterial strains, plasmids, and primers used in this study. [file 12934_2019_1152_MOESM3_ESM.docx]

Additional file 3. List of strains, plasmids and primers used in this study

| **Strain, plasmid, or primer** | Description | Source or reference |
| --- | --- | --- |
| Strains |  |  |
| *Escherichia coli* DH5α | *fhuA2 lac(del)U169 phoA glnV44 Φ80' lacZ(del)M15 gyrA96 recA1 relA1 endA1 thi- 1 hsdR17* | Takara Biotechnology Co., Ltd |
| *B.subtilis*168 | trpC2 | Laboratory collection |
| *B.subtilis*W800N | *npr*E *apr*E *epr* *bpr* *mpr* :: *ble* *npr*B :: *bsr* ∆*vpr wpr*A :: *hyg* cm :: neo; Neo^R^ | Laboratory collection |
| Plasmids |  |  |
| pDG1730 | Integration and shuttle plasmid, amyE integration site | Laboratory collection |
| pDG1730-CotC-treS | CotC-treS fusion gene, *amyE* integration site | This study |
| pDG1730-CotG-treS | CotC-treS fusion gene, *amyE* integration site | This study |
| pDG1730-CotC-treS-CotC-treS | CotC-treS-CotG-treS fusion gene, *amyE* integration site | This study |
| Primers |  |  |
| cotC-F | AAAACTGGTCTGATC*GGATCC*GATAAATCGTTTGGGCCGATG *BamH*I | This study |
| cotC-R | GGCTGGGTCATGTAGTGTTTTTTATGCTTTTTATACTCTACAA | This study |
| cotG-F | AAAACTGGTCTGATC*GGATCC*CGTAAAGCGGTAAATTGGATTGA *BamH*I | This study |
| cotG-R | CGGGCTGGGTCATTTTGTATTTCTTTTTGACTACCCAGC | This study |
| treS-R | CTGCAGGAATTCGAT*AAGCTT*TCAGTGGTGGTGGTGGTGGT *Hind*III | This study |
| cotC-treS-F-1 | AAACACTACATGACCCAGCCCGACCCG | This study |
| cotG-treS-F-1 | ATACAAAATGACCCAGCCCGACCCG | This study |
| cotC-treS-F-2 | AAAACTGGTCTGATC*GGATCC*GGTGGCGGTGGCTCGGGC | This study |
| cotC-treS-R-2 | TACCGCTTTACGTCAGTGGTGGTGGTGGTGGT | This study |
| cotG-treS-F-2 | ACCACTGACGTAAAGCGGTAAATTGGATTGA | This study |
| coG-treS-R-2 | CTGCAGGAATTCGATAAGCTTTCAGTGGTGGTGGTGGTGGT *Hind*III | This study |
| sleB-F | CGGGATCCCGGGGGATGATGTGGTCGAG *Bam*HI |  |
| sleB-R | ACTGACTCACTCAAAAATAACCCCCGCTACT |  |
| km-F | CGGGGGTTATTTTTGAGTGAGTCAGTCATCAGGAG |  |
| km-R | CGGGATCCCGGGTTGAGGCCGTTGAGCA *Bam*HI |  |
| cwlJ-F | CGGGATCCCGTTCTGAAGTAATGAAATATGATG *Bam*HI |  |
| cwlJ-R | CTATGGTGTGTGGGAAAAGCAGTGTGACTTAAATCT |  |
| zeo-F | TAAGTCACACTGCTTTTCCCACACACCATAGCTTCAA |  |
| zeo-R | CGGGATCCCGGTTGGTCTCCAGCTTGCAAA *Bam*HI |  |
