# Supplementary material for: Production of trehalose with trehalose synthase expressed and displayed on the surface of Bacillus subtilis spores
Source: Microb Cell Fact. 2019 Jun 3;18:100. doi: 10.1186/s12934-019-1152-7 (PMC6547511; doi:10.1186/s12934-019-1152-7)
Supplement: Supplementary file 7 — Additional file 7. Construction of recombinant B. subtilis WB800n with deletion of genes sleB and cwlJ. [file 12934_2019_1152_MOESM7_ESM.docx]

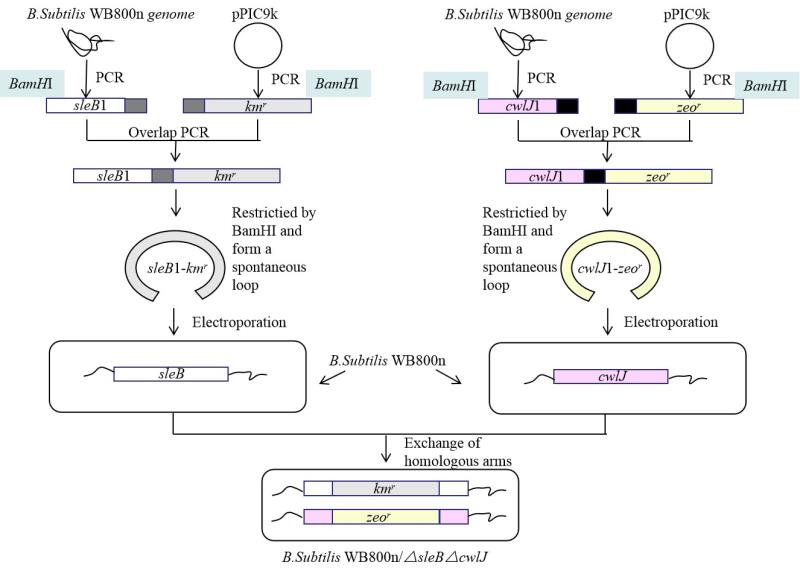


Additional file 7 Construction of recombinant *B. subtilis* W800N with the deletion in the *selB* and *cwlJ* genes.
